# NLRP3 inflammasome *via* IL-1β regulates PCSK9 secretion

**DOI:** 10.7150/thno.45939

**Published:** 2020-05-30

**Authors:** Zufeng Ding, Xianwei Wang, Shijie Liu, Sichang Zhou, Rajshekhar A Kore, Shengyu Mu, Xiaoyan Deng, Yubo Fan, Jawahar L Mehta

**Affiliations:** 1 Henan Key Laboratory of Medical Tissue Regeneration, Xinxiang Medical University, Xinxiang, China.; 2 Central Arkansas Veterans Healthcare System and Department of Internal Medicine, University of Arkansas for Medical Sciences, Little Rock, USA.; 3 Department of Neurological Surgery, Weill Cornell Medicine, New York, USA.; 4 Department of Pharmacology and Toxicology, College of Medicine, University of Arkansas for Medical Sciences, Little Rock, USA.; 5 Key Laboratory for Biomechanics and Mechanobiology of Ministry of Education, School of Biological Science and Medical Engineering, Beihang University, Beijing, China.; 6 Beijing Advanced Innovation Center for Biomedical Engineering; Beihang University, Beijing, China.

**Keywords:** PCSK9, NLRP3 inflammasome, IL-1β, MAPKs, atherosclerosis

## Abstract

**Background:** Both PCSK9 and NLRP3 inflammasome play important roles in atherogenesis. This study was designed to test the hypothesis that NLRP3 inflammasome via IL-1β induces PCSK9 secretion. The inter-twined relationship between NLRP3 inflammasome, IL-1β and PCSK9 may be relevant in atherogenesis.

**Methods:** We studied NLRP3 inflammasome-mediated PCSK9 secretion in mouse peritoneal macrophages and in a variety of tissues, such as liver, kidney and small intestine. Macrophages were derived from wild-type (WT) and a variety of gene deletion mice to define the mechanistic basis of NLRP3 inflammasome -mediated PCSK9 secretion. Additional studies were performed in high-fat diet fed mice.

**Results:** We observed that NLRP3 and its downstream signals ASC, Caspase-1, IL-18, and IL-1β all participate in PCSK9 secretion. IL-1β seems to be more important than IL-18 in the induction of PCSK9 secretion. Further, there appears to be significant involvement of MAPKs in this process. Lastly, we observed that mice fed high fat diet have high expression of NLRP3 and a greater secretion of PCSK9 than mice fed a standard diet, and this increased secretion of PCSK9 in high fat diet-fed mice was attenuated in *IL-1β^-/-^* mice.

Conclusions: This study based on extensive in vitro and in vivo data provides evidence that NLRP3 inflammasome via IL-1β plays an important role in determining PCSK9 secretion, particularly in the presence of high-fat diet.

## Introduction

Atherosclerotic cardiovascular diseases remain major public health issues in the US, and rapidly becoming a major issue worldwide 1. Although much is known about the steps involved in atherogenesis, efforts are being made to define new biological processes that are responsible for the development of atherosclerosis. One of the new processes being recognized is the role that PCSK9 plays in atherogenesis and its clinical manifestations. Predominantly expressed in liver, kidney and small intestine, PCSK9 binds with LDLr on the surface of the cells, resulting in degradation of LDLr and increase in LDL-cholesterol levels 2,3. PCSK9 monoclonal antibodies have been approved for clinical use. These agents reduce major adverse cardiovascular events 4,5. PCSK9 silencing has also been shown to induce a drastic and sustained lowering of LDL-C levels 3.

Though PCSK9 is mainly secreted by liver and small intestine, recent studies show that PCSK9 is also expressed in vascular smooth muscle cells (SMCs) and endothelial cells (ECs) 6, and macrophages 7. The expression of PCSK9 is particularly evident when these cells are exposed to inflammatory stimuli, such as lipopolysaccharide (LPS) Atherosclerotic tissues show intense staining for PCSK9 6. Ferri et al. have reported that PCSK9 sustains VSMC synthetic phenotype, and their proliferation and migration, which play a pro-atherogenic role in the arterial wall 8. Other studies by their group have shown that PCSK9 levels in plasma are related to interferon-γ 9, air pollution 9 and vascular stiffness 10.

NLRP3 inflammasome is a caspase-1-activating complex that is involved in IL- 1β and IL-18 secretion 11. The NLRP3 inflammasome is comprised of NLRP3, ASC, and caspase-1, which is assembled in response to bacterial LPS and is activated by diverse stimuli such as ox-LDL 10-12. Since LPS induces both NLRP3 inflammasome activation and PCSK9 secretion in macrophages 12,13, we postulated that the NLRP3 inflammasome may play a role in the regulation of PCSK9 secretion. This study was designed to test this postulate.

Although several pro-inflammatory cytokines, such as IL-6, MCP-1, IFN-γ and TNF-α, regulate PCSK9 secretion, we focused on the possible role of NLRP3 inflammasome activation in regulating PCSK9 secretion in this study. For the first time, we show that NLRP3 inflammasome activation upregulates PCSK9 secretion. We also show that IL-1β, a downstream signal in NLRP3 inflammasome activation, and MAPKs play key roles in this process.

## Results

### Macrophages secrete large amount of PCSK9 via NLRP3 inflammasome activation

**Figure 1:**
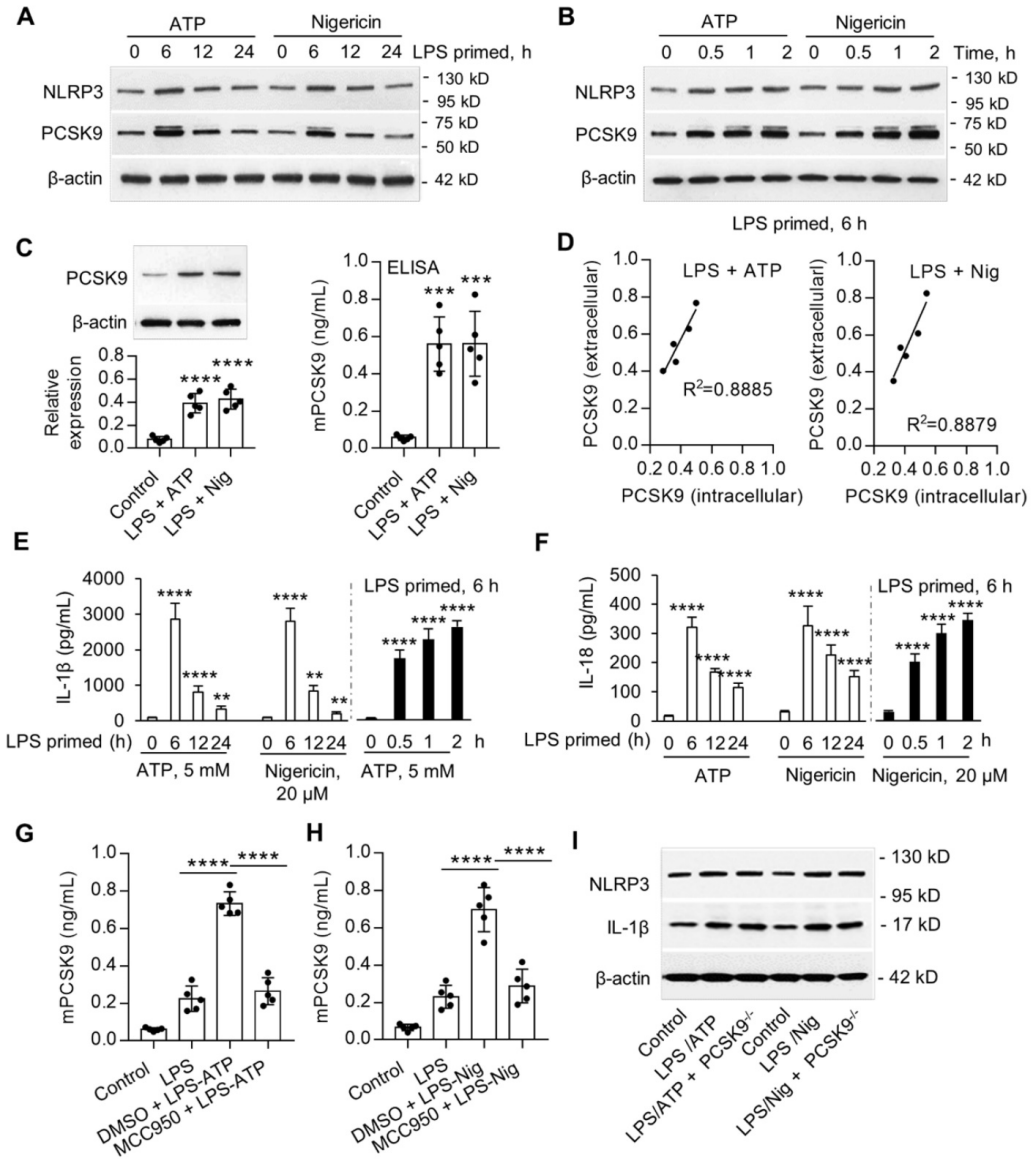
** NLRP3 inflammasome induces PCSK9 expression.** (**A**) and (**B**) NLRP3 and PCSK9 expression in LPS primed macrophages treated with ATP or nigericin. (**C**) PCSK9 expression (intracellular, measured by western blot) and its secretion (extracellular, measured by ELISA) from LPS-primed MPMs treated with ATP or nigericin. (**D**) Pearson correlation coefficient (R) analysis for the correlation between intracellular expression and its release based on the results from panels **C**. (**E**) and (**F**) Release of IL-1β and IL-18 from LPS-primed MPMs treated with ATP or nigericin. Cytokine levels were normalized to that in DMSO-treated cells. (**G**) and (**H**) Effect of NLRP3 inflammasome specific inhibitor MCC950 on PCSK9 secretion from LPS-primed MPMs treated with ATP or nigericin. (**I**) Effect of PCSK9 gene deletion on expression of NLRP3 and IL-1β. Western blots in each group were performed with the same protein concentration and the same film exposure time. Data represent the mean ± SD of independent experiments (n=5 mice per genotype), each performed in triplicate. *P<0.05, **P<0.01, ***P<0.001, ****P<0.0001 vs. indicated group. MPMs: mouse peritoneal macrophages.

Previous studies have shown that ATP and nigericin activate NLRP3 inflammasome 14. In preliminary studies, we established that priming of mouse peritoneal macrophages (MPMs) with LPS was necessary for the expression of NLRP3 inflammasome. Here, we examined if ATP and nigericin would induce PCSK9 secretion besides NLRP3 expression in wild-type (WT) mouse peritoneal macrophages (MPMs). As shown in **Figure 1A** and **1B**, ATP and nigericin not only induced NLRP3 inflammasome in MPMs primed with LPS but also induced PCSK9 expression simultaneously. The expression of NLRP3 inflammasome and PCSK9 in response to ATP and nigericin was maximal at 6 h of LPS priming (**Figure 1A**). Therefore, in subsequent experiments we used MPMs that had been primed with LPS for 6 h. As shown in **Figure 1B**, expression of both NLRP3 inflammasome and PCSK9 in response to ATP and nigericin was also time-dependent**.** These observations of simultaneous induction of NLRP3 inflammasome and PCSK9 in response to ATP and nigericin suggested a link between NLRP3 inflammasome activation and PCSK9 secretion.

To study if there is a concordance between PCSK9 protein expression intracellularly and its extracellular release, we measured protein in the MPMs (by western blot) and in the supernatants of MPMs (by ELISA). As shown in **Figure 1C**, ATP and nigericin both significantly increased intracellular PCSK9 expression and its extracellular release. Importantly, there was a significant correlation between intracellular expression and extracellular release (**Figure 1D**).

We posited that cytokines such as IL-1β and IL-18 released during NLRP3 inflammasome induction 15 may regulate PCSK9 secretion in MPMs. Indeed, both ATP and nigericin were found to induce the release of IL-1β and IL-18 in LPS-primed MPMs in a time-dependent manner (**Figure 1E** and** 1F**). As with the expression of NLRP3 and PCSK9, release of IL-1β and IL-18 was maximal at 6 h of LPS priming, and then decreased sharply.

Next, we studied if NLRP3 inflammasome activation is involved in PCSK9 secretion. MPMs were treated with the DMSO or with the NLRP3 inhibitor MCC950. As shown in **Figure 1G** and **IH**, compared with control, MCC950 treatment inhibited ATP- and nigericin- induced PCSK9 secretion. To clarify if PCSK9 can regulate the expression of NLRP3 and IL-1β, we used MPMs isolated from PCSK9 gene deletion mice. As shown in **Figure 1I**, PCSK9 gene deletion had almost no effect on the expression of both NLRP3 and IL-1β, indicating that PCSK9 expression is downstream of NLRP3 and IL-1β.

### ASC, Caspase-1, IL-1β and IL18 are involved in NLRP3 inflammasome-mediated PCSK9 expression

**Figure 2:**
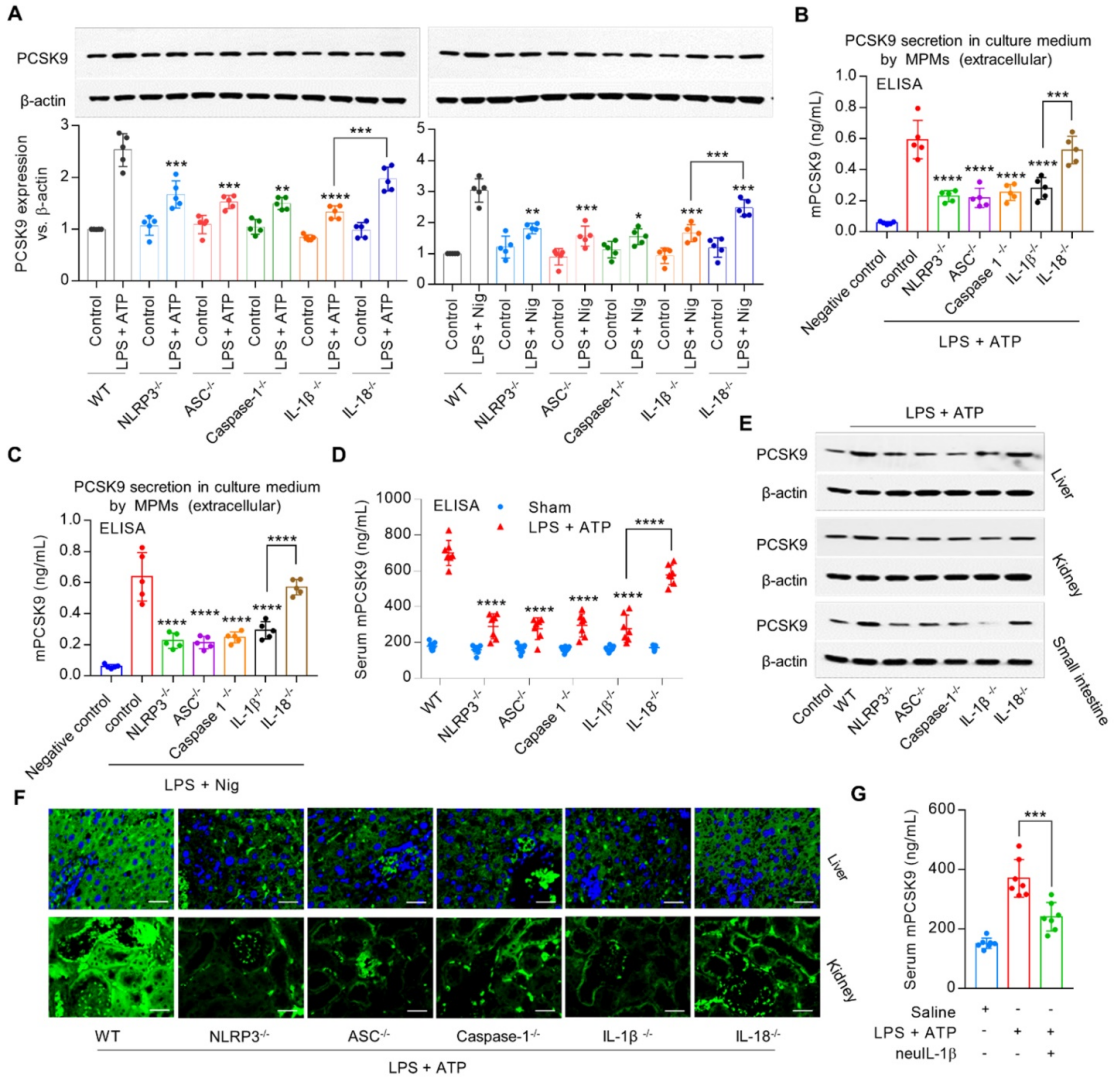
** NLRP3 inflammasome gene deletion reduces PCSK9 expression.** (**A**) to (**C**) PCSK9 expression in MPMs at both intracellular and extracellular levels. Western blot quantification is shown as fold change vs. WT control (considered as baseline=1). *P<0.05, **P<0.01, ***P<0.001, ****P<0.0001 vs. control or indicated group. (**D**) PCSK9 secretion in serum. (**E**) and (**F**) PCSK9 expression in different tissues. Mice were given LPS and ATP by intraperitoneal route 6 h before collection of blood. Inhibition of PCSK9 secretion is much less in IL18^-/-^ mice than in IL-1β^-/-^ mice. ****P<0.0001 vs. WT in LPS + ATP group or indicated group. Scale bar: 20 µm. (**G**) PCSK9 secretion in serum with or without neuIL-1β pretreatment. Western blots in each group were performed with the same protein concentration and the same film exposure time. Data represent the mean ± SD of independent experiments (n=5 mice per genotype in cell experiments and n=7 mice per genotype in animal experiments), each performed in triplicate.

To investigate the role of signals downstream of NLRP3 inflammasome in PCSK9 secretion, we used MPMs from *NLRP3^-/-^, ASC^-/-^, Caspase-1^-/-^, IL-1β^-/-^* and* IL18^-/-^* mice. We observed that both intracellular (**Figure 2A**) and extracellular PCSK9 expression (**Figure 2B** and **2C**), and serum PCSK9 level (**Figure 2D**) were much less from *NLRP3^-/-^, ASC^-/-^,* and *Caspase-1^-/-^* mice as well as from *IL-1β^-/-^* and* IL-18^-/-^* mice compared with MPMs from WT mice.

PCSK9 is highly expressed in liver, kidney and small intestine, and to a much smaller extent in aorta, heart, spleen, lung and brain 3. Consistent with this concept, our data based on western blotting (**Figure 2E** and **Supplemental Figure 1B**) and immunofluorescent staining (**Figure 2F**) showed that PCSK9 was highly expressed in liver, kidney and small intestine. Interestingly, tissues from *NLRP3^-/-^, ASC^-/-^, Caspase-1^-/-^*,* IL-1β^-/-^* and* IL18^-/-^* mice revealed markedly lower PCSK9 expression compared with WT mice*.*

It is noteworthy that *IL-18^-/-^* mice showed a small (≈15%) decrease in PCSK9 secretion; in contrast, *IL-1β^-/-^* mice revealed a large (≈53%) decrease in PCSK9 secretion compared with that in the WT mice (**Figure 2D**), suggesting that IL-1β may have a more robust effect on PCSK9 release as compared with IL-18 following exposure to inflammatory stimuli. Further experiments showed that pretreatment with mouse anti-IL-1β neutralizing monoclonal antibody (neuIL-1β) markedly decreased LPS with ATP- induced PCSK9 secretion in serum (**Figure 2G**). Accordingly, we chose to focus on IL-1β in the regulation of PCSK9 secretion in subsequent experiments.

### MAPKs are involved in IL-1β-induced PCSK9 secretion

**Figure 3:**
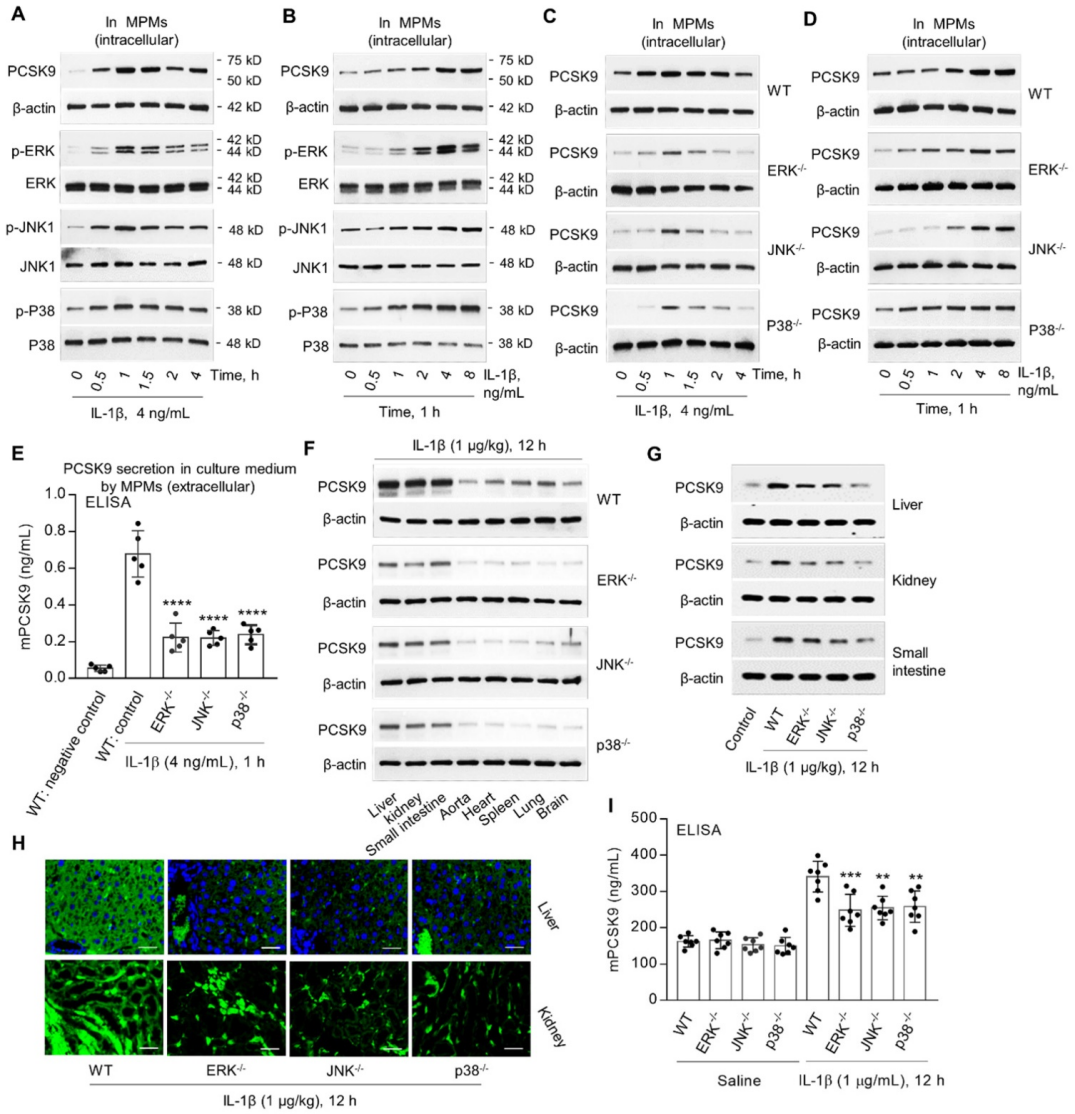
** MAPKs are involved in IL-1β-induced PCSK9 secretion.** (**A**) and (**B**) IL-1β and PCSK9 secretion and MAPKs expression. (**C**) and (**D**) PCSK9 expression in WT mice, ERK^-/-^, JNK^-/-^ and P38^-/-^ mice. (**E**) ELISA analysis for PCSK9 secretion in MPM culture medium. ****P<0.0001 vs. WT control. (**F**) to (**H**) PCSK9 expression in different tissues from ERK^-/-^, JNK^-/-^ and P38^-/-^ mice measured by western blot or immunofluorescent staining. Scale bar: 20 µm. (**I**) Serum mPCSK9 levels in WT, ERK^-/-^, JNK^-/-^ and P38^-/-^ mice. ****P<0.0001 vs. WT + saline; ^++^P<0.01, ^++^P<0.001 vs. WT. Western blots in each group were performed with the same protein concentration and the same film exposure time. Data represent the mean ± SD of independent experiments (n=5 mice per genotype in cell experiments and n=7 mice per genotype in animal experiments), each performed in triplicate. MPMs: mouse peritoneal macrophages.

MAPKs, such as ERK, JNK and P38, are involved in IL-1β signaling 16,17. We posited that IL-1β-mediated PCSK9 secretion might involve MAPKs. In the present studies, we observed that IL-1β treatment of MPMs from WT mice was associated with increased expression of p-ERK, p-JNK and p-P38 as well as PCSK9 in a dose- and time-dependent manner (**Figure 3A** and **3B**). Notably, there was no change in unphosphorylated ERK, JNK and P38. Importantly, PCSK9 expression was much less in MPMs from *ERK^-/-^, JNK^-/-^*and* P38^-/-^* mice compared with MPMs from WT mice (**Figure 3C** and **3D**). Further, ELISA showed that extracellular PCSK9 secretion (**Figure 3E**) was also lower in *ERK^-/-^, JNK^-/-^*and* P38^-/-^* mice compared with WT group.

To further confirm the role of ERK, JNK and P38 in IL-1β - induced PCSK9 secretion, we measured PCSK9 expression and secretion in different tissues. We observed that PCSK9 expression in different tissues- liver, kidney, small intestine, aorta, heart, spleen, lung and brain was much less in *ERK^-/-^, JNK^-/-^*and* P38^-/-^* mice as compared with WT mice (**Figure 3F to 3H**). PCSK9 secretion in blood was also much less in *ERK^-/-^, JNK^-/-^*and* P38^-/-^* mice as compared with WT mice, all given IL-1β (1 µg/kg) (**Figure 3I**).

It is well-known that ERK signaling regulates insulin sensitivity 17. We, therefore, investigated the role of insulin in regulating PCSK9 expression in macrophages. As shown in **Supplemental Figure 1C**, insulin induced PCSK9 expression in a dose- and time- dependent manner, indicating that insulin was indeed involved in ERK-mediated PCSK9 expression.

### IL-1β -PCSK9 interaction in high fat diet (HFD-C) diet fed mice

**Figure 4:**
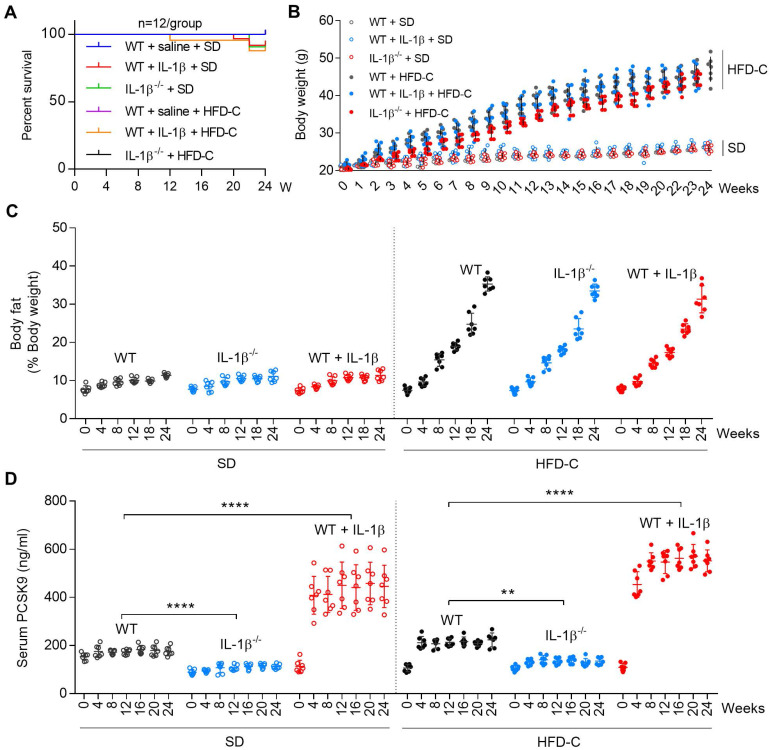
** IL-1β induces PCSK9 secretion in mice.** (**A**) Survival rate for different groups of mice fed standard diet (SD) or high fat diet (HFD-C). (**B**) and (**C**) Body weight and body fat of WT and IL-1β^-/-^ mice given SD or HFD-C for 24 weeks. All mice were given recombinant IL-1β (**D**) Serum PCSK9 levels in WT and IL-1β^-/-^ mice. Data represent the mean ± SD of several independent experiments (n=5 mice per genotype in cell experiments and n=7 mice per genotype in animal experiments), each performed in triplicate. **P<0.001, ***P<0.001, ****P<0.0001 vs. indicated group.

Since both NLRP3 and IL-1β are activated in mice fed with high fat diet 18, we studied if IL-1β can regulate PCSK9 secretion in mice fed HFD-C diet. Groups of WT and *IL1β^-/-^* mice were fed SD or HFD-C. To maintain heightened state of inflammation, some WT mice were administered recombinant mouse IL-1β intravenously. The survival rate of mice was not affected by IL-1β alone or HFD-C (**Figure 4A**). Body weight and body fat composition also increased similarly over the 24-week period in all mice groups (**Figure 4B** and **4C**).

Most interestingly, serum PCSK9 levels were lower in *IL-1β^-/-^* mice as compared with WT mice (fed SD or HFD-C) throughout the 24-week period. Serum PCSK9 levels were exacerbated by HFD-C in WT as well as in *IL1β^-/-^* mice, The PCSK9 levels increased markedly when given IL-1β (P<0.05 vs. mice fed SD or HFD-C) (**Figure 4D**).

### IL-1β -PCSK9 interaction regulates LDLr expression and LDL cholesterol (LDL-C) levels

**Figure 5:**
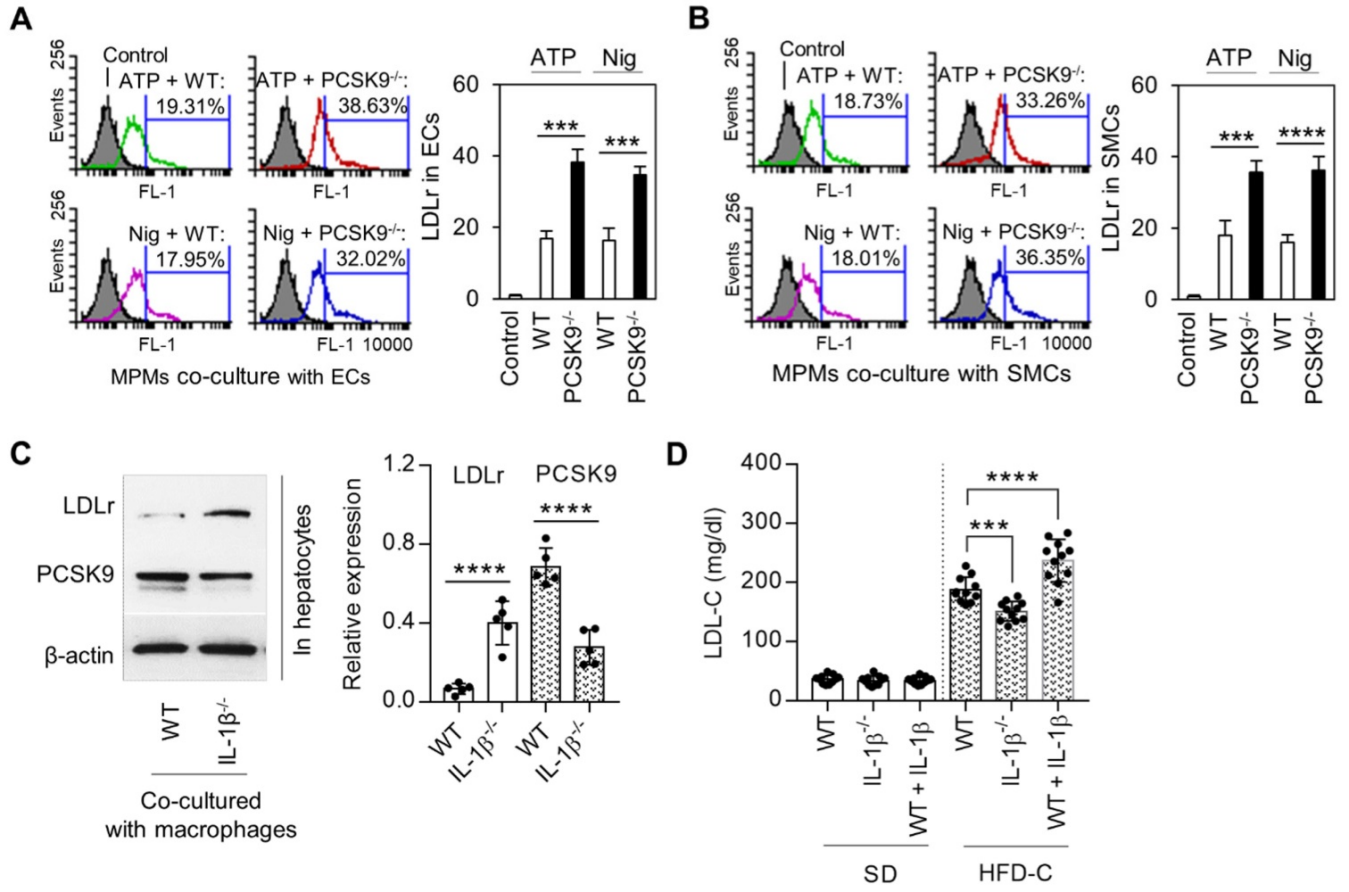
** IL-1β mediated PCSK9 and its role in regulating of LDLr expression and LDL-C level.** (**A**) and (**B**) Flow cytometry analysis for LDLr expression in endothelial cells (ECs) or smooth muscle cells (SMCs) co-cultured with mice peritoneal macrophages (MPMs) from both WT and PCSK9^-/-^ mice. (**C**) Expression of LDLr and PCSK9 in primary hepatocytes that co-cultured with LPS-primed macrophages. MPMs were primed with 100 ng/ml LPS for 6h, followed by incubation with 5 mM ATP or 20 μM nigericin for 1h. (**D**) Serum LDL-C levels in WT mice, WT mice treated with IL-1β, and IL-1β^-/-^ mice at 24 weeks fed SD or HDF-C. Bar graphs represent data compiled from three independent experiments (n=5 mice per genotype in cell experiments and n=11 mice per genotype in animal experiments), shown as mean ± SD. *P<0.05, **P<0.01, ***P<0.001, ****P<0.0001 vs. indicated group.

Macrophages along with ECs and SMCs play a key role in atherogenesis. We investigated if PCSK9 secreted by MPMs modulated the expression of LDLr in ECs and SMCs. For this purpose, we measured LDLr expression on mouse aortic ECs and SMCs that had been co-cultured with MPMs. As shown in **Figure 5A** and **5B**, LDLr expression was significantly decreased (~50%) in both ECs and VSMCs co-cultured with MPMs obtained from WT mice and treated with ATP and nigericin. Importantly, compared with WT MPMs, *PCSK9^-/-^* MPMs markedly enhanced LDLr expression in both ECs and SMCs.

In order to clarify the role of macrophage-secreted IL-1β in the regulation of LDLr in concert with PCSK9 expression in hepatocytes, the major source for blood PCSK9, we co-cultured primary hepatocytes with MPMs isolated from WT and *IL-1β^-/-^* mice. As shown in **Figure 5C**, LDLr expression in hepatocytes was much higher in *IL-1β^-/-^* mouse-derived MPMs compared WT mouse-derived MPMs. Of note, PCSK9 expression was much lower in hepatocytes co-cultured with *IL-1β^-/-^* mouse-derived MPMs compared WT mouse-derived MPMs.

Finally, we analyzed LDL-C levels at 24 weeks of SD or HFD-C administration. As shown in **Figure 5D**, there was almost no difference in LDL-C levels between different groups of mice fed SD. However, IL-1β treatment enhanced LDL-C level in mice fed HFD-C diet; this increase was markedly attenuated in *IL-1β^-/-^* mice fed HFD-C.

## Discussion

This study based on extensive in vitro and in vivo experiments shows that NLRP3 inflammasome induces PCSK9 in macrophages as well as in a host of tissues including liver, small intestine and kidney. This study also shows that the induction of PCSK9 by NLRP3 inflammasome is dependent on IL-1β release.

Inflammasomes are a group of cytosolic protein complexes that are formed to mediate host immune responses to microbial infection and cellular damage. NLRP3 inflammasome assembly triggers proteolytic cleavage of dormant procaspase-1 into active caspase-1 and produces biologically active IL-1β and IL-18 19. A number of stimuli, such as LPS, ATP, nigericin, microbial products, high fat diet and particulate matter, have been shown to activate NLRP3 inflammasome 20,21. In keeping with these previous observations, we showed that ATP as well as nigericin, in LPS-primed macrophages, induced the expression of NLRP3 inflammasome and simultaneously enhanced PCSK9 expression. All steps downstream of NLRP3 inflammasome were involved in PCSK9 expression. This was confirmed with the use of macrophages from *ASC, Caspase-1* and* IL-1β* gene deletion mice. This concept was further verified by measuring PCSK9 levels in serum of different groups of mice as well as protein expression in different organs that normally express and release PCSK9.

It is of note that while the secretion of both IL-1β and IL-18 from macrophages in response to ATP or nigericin was significant, IL-18 gene deletion had much less inhibitory effect on PCSK9 secretion compared with IL-1β deletion, indicating that IL-1β is the major mediator for PCSK9 secretion.

Although PCSK9 is mostly expressed in the liver, kidney and small intestine, other tissues such as heart, artery, brain and pancreas also express and secrete PCSK9. Macrophages in the basal state express small amounts of PCSK9, but PCSK9 expression increases several-fold in inflammatory states 11,13. As one of the major immune cells activated in the pro-inflammatory milieu, macrophages are involved in the detection, phagocytosis and destruction of bacteria and other harmful organisms 15. In this study, we show that NLRP3 inflammasome in macrophages induces PCSK9 expression mainly via IL-1β. Macrophages not only produce IL-1β and IL-18, but also produce ROS and nitroso compounds that can kill phagocytosed bacteria 19. Hence, PCSK9 released from macrophages via IL-1β may be relevant in atherogenesis. Of note, we have previously shown that ROS are necessary for PCSK9 expression and release 6.

Our study also shows that MAPKs play important roles in IL-1β-mediated PCSK9 secretion, and may serve as the major mechanistic link between NLRP3 inflammasome and PCSK9 expression. These conclusions are based on studies in isolated macrophages as well as in a variety of tissues, including liver, kidney, small intestine, aorta, heart, spleen, lung and brain. The important role of MAPKs in NLRP3-induced PCSK9 generation was confirmed with the use of mice with specific gene deletion.

Elevated levels of LDL-cholesterol induce atherosclerosis in susceptible animal species as well as in humans 22,23. An inflammatory state is a hallmark of atherosclerotic lesions, and atherosclerotic tissues reveal high expression of NLRP3 inflammasome 14. Our recent studies show that atherosclerotic tissues also express PCSK9 6. In the present study, we show that HFD-C results in robust secretion of PCSK9 in serum, and this PCSK9 secretion is dependent on IL-1β upregulation since PCSK9 expression was markedly lower in *IL-1β^ -/-^* mice given the same HFD-C. These observations provide a potent link between IL-1β and PCSK9 in pro-atherosclerotic and pro-inflammatory milieu.

Based on the data reported here, we conclude that NLRP3 inflammasome activation via IL-1β is a powerful inducer for PCSK9 secretion in both macrophages and tissues. It is likely that liver-secreted PCSK9 is the main source of circulating PCSK9 in mice given HFD-C. However, macrophage secreted PCSK9 via its local effect in the atherosclerotic plaque may also play an important role in atherogenesis 13.

Interestingly, Giunzioni et al. 13 reported that PCSK9 increases the expression of pro-inflammatory cytokines, such as TNFα, IL-1β, IL-10 and Arg1 in LPS-stimulated macrophages. Ricci et al. 24 observed that PCSK9 induces a significant increase in IL-1β, IL-6, TNF-α, CXCL2, and MCP1 mRNA in macrophages. Ruscica et al. 25 found that TNF-α induced PCSK9 via regulating suppressor of cytokine signaling 3 (SOCS3). Jeong et al. 26 reported that expression of nuclear forms of sterol-regulatory element binding protein-1 (SREBP-1) and SREBP-2 dramatically increased the promoter activity of PCSK9. SREBP activates NLRP inflammasome in a variety cells including macrophages 27. Therefore, it is reasonable to conclude that SREBP-1 and SREBP-2 activate PCSK9 via NLRP3 inflammasome. These data taken together suggest a bidirectional interaction between pro-inflammatory cytokines and PCSK9, and the key role of NLRP3 inflammasome- which may be relevant in atherosclerosis.

The role of macrophage-derived PCSK9 in regulating serum concentrations of PCSK9 remains to be determined. However, our observations of similarly elevated serum cholesterol concentrations in WT and IL-1β^-/-^ mice fed HFD-C suggest that IL-1β-derived PCSK9 may have a limited effect on the determination of serum cholesterol concentrations, and yet the PCSK9 expression was markedly elevated in activated MPMs and less so in MPMs from IL-1β^-/-^ mice.

In summary, this study based on the use of a host of mice species provides first definitive data on the link between NLRP3 inflammasome and PCSK9 secretion. The NLRP3 inflammasome- PCSK9 signaling involves ASC, Caspase-1 and IL-1β. IL-1β secretion appears to be the mechanistic link between NLRP3 inflammasome. We did not identify any difference in PCSK9 secretion and accumulation of LDL-c between mice fed SD and given IL-1β^-/-^ mice; however, feeding HFD-C dramatically increased LDL- levels in all groups, indicating that high fat western diet is much more important than IL-1β status.

The critical role of IL-1β in patients with ischemic heart disease is supported by a recent study that showed an almost 25% reduction in cardiovascular events with the use of IL-1β antibody 28. Other studies showed a significant reduction in cardiovascular events in patients treated with PCSK9 monoclonal antibodies 3-5.

Several recent studies have shown a link between NLRP3, IL-1β and PCSK9 in inflammatory states other than the cardiovascular system 29,30. The present study provides definitive evidence for the link between NLRP3 inflammasome activation, IL-1β and PCSK9 expression/release in macrophages and other organs, and sheds light on their potential contribution to atherogenesis.

## Materials and methods

### Animals

C57BL/6 wild-type (controls), *PCSK9^-/-^, NLRP3^-/-^, Caspase^-/-^, IL-1β^-/-^, IL18^-/-^, ERK^-/-^, JNK^-/-^ and P38^-/-^ (P38^AF^)* mice were purchased from the Jackson Laboratory (Sacramento, CA) and housed in the animal care facility of our institution. ASC^-/-^ mice were obtained from Millennium Pharmaceuticals (Cambridge, MA). The data for gene knockout validation are shown in **Supplemental Figure 1A**. All mice species were generated on C57BL/6 background. All experimental procedures were performed in accordance with protocols approved by the Institutional Animal Care and Usage Committee, and conformed to the Guidelines for the Care and Use of Laboratory Animals published by the US National Institutes of Health. Only male mice approximately 6 weeks of age were used in this study.

To induce cytokine production, mice were given LPS from Escherichia coli 0111:B4 (Sigma) intraperitoneally, dose 25 mg/kg body weight. Six hours later, ATP (50 mM, adjusted to pH 7.0) was administered intraperitoneally of dose 10 µL/g body weight. One hour later, mice were euthanatized and blood was collected. To investigate the role of IL-1β in the regulation of PCSK9 secretion, another group of mice were administered saline or mouse recombinant IL-1β (1 μg/kg) or mouse IL-1β neutralizing antibody (1 mg/mouse, InvivoGen) 12 h prior to sacrifice of the animals.

### High fat and cholesterol diet administration

Groups of WT and *IL-1β^-/-^* mice at 6 weeks of age were fed either a standard diet (SD) or high fat with cholesterol diet (HFD-C, 20% anhydrous milk fat, 0.2% cholesterol, Bio-Serv, NJ) and maintained on a 12 h light-dark cycle. Whole body fat mass was measured by using the Minispec mq10 NMR analyzer (Brucker Optics, Woodlands, TX) according to the supplied protocol. To maintain a heightened state of inflammation, some WT mice were administered recombinant IL-1β (10 ng/kg) every 3 days. At 24 weeks of SD or HFD-C, mice were sacrificed and blood was collected.

### Isolation of peritoneal macrophages

Mice were administered 1 ml of 4% thioglycolate in PBS intraperitoneally. Three days later, peritoneal cells were collected and incubated in DMEM/F12 supplemented with 10% fetal bovine serum for 4 hours. Cells were then incubated at 37 °C for 6h and washed with PBS to remove the non-adherent cells. The remaining adherent cells were used as the peritoneal macrophages described in the experiments. Unless otherwise indicated, the macrophages were primed with 100 ng/ml LPS from Escherichia coli 0111:B4 (Sigma) for 6 h before stimulation with 5 mM ATP or 20 μM nigericin for 1h. To study the role NLRP3 inflammasome in regulation of PCSK9 secretion, NLRP3 inflammasome inhibitor MCC950 (InvivoGen, San Diego, CA) at 1 μM was pretreated to macrophages before LPS treatment.

### ELISA for PCSK9, IL-1β, IL-18

PCSK9 secretion was measured in serum samples and cultured cell media using PCSK9 ELISA kit (MBL International, Nagoya, Japan). IL-1β and IL-18 were measured in mouse serum samples and cell culture media with mouse IL-1β and IL-18 ELISA kit (Abcam, San Francisco, CA). All assays were performed according to the manufacturer's instructions.

### Western blotting

Proteins from tissues and cells were purified with RIPA Lysis Buffer System (Santa Cruz, CA), and loaded onto 12% Mini-PROTEAN® TGX™ Precast Gel (Bio-rad, CA) for electrophoresis. The size-separated proteins will be then transferred to Hybond ECL Nitrocellulose Membranes (GE Healthcare, NJ). After blocking with 5% BSA buffer for 1 h, the membranes were incubated with primary antibody at 1:1000 dilution overnight at 4 °C. After washing with PBS containing 0.1% Tween-20, membranes were incubated with secondary antibody for 1 h and signals were detected with Pierce ECL Western Blotting Substrate (Thermo Scientific, IL). Intensity quantification of the bands were obtained with Image J software and normalized to β-actin.

Antibodies directed at PCSK9 (Cat. ab31762), NLRP3 (Cat. ab214185) and IL-1β (Cat. ab9722) were purchased from Abcam (San Francisco, CA). Antibody directed at p-JNK1 was purchased from Thermo Scientific (Cat. PA5-37698) and JNK1 antibody was purchased from Santa Cruz (Cat. sc-1648). Antibodies directed at p-ERK (Cat. 8544), ERK (Cat. 4695), p-P38 (Cat. 4511), P38 (Cat. 8690) and Cleaved Gasdermin D (Cat. 50928) were purchased from Cell signaling (Danvers, MA).

### Statistical analysis

Data are presented as means ± SD, representative of five mice per genotype (n=5 in cell experiments) or seven mice per genotype (n=7 in animal experiments) from three independent experiments. Significance between two groups was examined by unpaired t-test. Multiple comparisons were analyzed by one-way ANOVA, followed by Tukey post hoc comparisons test. All analyses were performed by GraphPad Prism version 7.00 (GraphPad Software, SanDiego, CA, USA). A P value of <0.05 was considered statistically significant.

## Supplementary Material

Supplementary figures and tables.

## References

[1] Benjamin EJ, Muntner P, Alonso A (2019). American Heart Association Council on Epidemiology and Prevention Statistics Committee and Stroke Statistics Subcommittee. Heart Disease and Stroke Statistics-2019 Update: A report from the American Heart Association. Circulation.

[2] Seidah NG, Abifadel M, Prost S, Boileau C, Prat A (2017). The proprotein convertases in hypercholesterolemia and cardiovascular diseases: emphasis on proprotein convertase subtilisin/kexin 9. Pharmacol Rev.

[3] Macchi C, Sirtori CR, Corsini A, Santos RD, Watts GF, Ruscica M (2019). A new dawn for managing dyslipidemias: The era of rna-based therapies. Pharmacol Res.

[4] Sever P, Gouni-Berthold I, Keech A, Giugliano R, Pedersen TR, Im K (2020). LDL-cholesterol lowering with evolocumab, and outcomes according to age and sex in patients in the FOURIER Trial. Eur J Prev Cardiol.

[5] Sabatine MS, Giugliano RP, Keech AC, Honarpour N, Wiviott SD, Murphy SA (2017). FOURIER Steering Committee and Investigators. Evolocumab and clinical outcomes in patients with cardiovascular disease. N Engl J Med.

[6] Ding Z, Liu S, Wang X, Deng X, Fan Y, Sun C (2015). Hemodynamic shear stress via ROS modulates PCSK9 expression in human vascular endothelial and smooth muscle cells and along the mouse aorta. Antioxid Redox Signal.

[7] Adorni MP, Cipollari E, Favari E, Zanotti I, Zimetti F, Corsini A (2017). Inhibitory effect of PCSK9 on Abca1 protein expression and cholesterol efflux in macrophages. Atherosclerosis.

[8] Ferri N, Marchianò S, Tibolla G, Baetta R, Dhyani A, Ruscica M (2016). PCSK9 knock-out mice are protected from neointimal formation in response to perivascular carotid collar placement. Atherosclerosis.

[9] Macchi C, Ferri N, Favero C, Cantone L, Vigna L, Pesatori AC (2019). Long-term exposure to air pollution raises circulating levels of proprotein convertase subtilisin/kexin type 9 in obese individuals. Eur J Prev Cardiol.

[10] Ruscica M, Ferri N, Fogacci F, Rosticci M, Botta M, Marchiano S, et al; Brisighella Heart Study Group (2017). Circulating levels of proprotein convertase subtilisin/kexin type 9 and arterial stiffness in a large population sample: data from the Brisighella Heart Study. J Am Heart Assoc.

[11] Latz E, Xiao TS, Stutz A (2013). Activation and regulation of the inflammasomes. Nat Rev Immunol.

[12] Ding Z, Liu S, Wang X, Dai Y, Khaidakov M, Deng X (2014). LOX-1, mtDNA damage, and NLRP3 inflammasome activation in macrophages: implications in atherogenesis. Cardiovasc Res.

[13] Giunzioni I, Tavori H, Covarrubias R, Major AS, Ding L, Zhang Y (2016). Local effects of human PCSK9 on the atherosclerotic lesion. J Pathol.

[14] Coll RC, Robertson AA, Chae JJ, Higgins SC, Muñoz-Planillo R, Inserra MC (2015). A small-molecule inhibitor of the NLRP3 inflammasome for the treatment of inflammatory diseases. Nat Med.

[15] He Y, Hara H, Núñez G (2016). Mechanism and regulation of NLRP3 inflammasome activation. Trends Biochem Sci.

[16] Wuyts WA, Vanaudenaerde BM, Dupont LJ, Demedts MG, Verleden GM (2003). Involvement of p38 MAPK, JNK, p42/p44 ERK and NF-kappaB in IL-1beta-induced chemokine release in human airway smooth muscle cells. Respir Med.

[17] Zhang W, Thompson BJ, Hietakangas V, Cohen SM (2011). MAPK/ERK signaling regulates insulin sensitivity to control glucose metabolism in Drosophila. PLoS Genet.

[18] Song W, Wei L, Du Y, Wang Y, Jiang S (2018). Protective effect of ginsenoside metabolite compound K against diabetic nephropathy by inhibiting NLRP3 inflammasome activation and NF-κB/p38 signaling pathway in high-fat diet/streptozotocin-induced diabetic mice. Int Immunopharmacol.

[19] Baroja-Mazo A, Martín-Sánchez F, Gomez AI, Martínez CM, Amores-Iniesta J, Compan V (2014). The NLRP3 inflammasome is released as a particulate danger signal that amplifies the inflammatory response. Nat Immunol.

[20] Strandberg L, Verdrengh M, Enge M, Andersson N, Amu S, Onnheim K (2009). Mice chronically fed high-fat diet have increased mortality and disturbed immune response in sepsis. PLoS One.

[21] Duewell P, Kono H, Rayner KJ, Sirois CM, Vladimer G, Bauernfeind FG (2010). NLRP3 inflammasomes are required for atherogenesis and activated by cholesterol crystals. Nature.

[22] Pirillo A, Bonacina F, Norata GD, Catapano AL (2018). The Interplay of lipids, lipoproteins, and immunity in atherosclerosis. Curr Atheroscler Rep.

[23] Seo T, Qi K, Chang C, Liu Y, Worgall TS, Ramakrishnan R (2005). Saturated fat-rich diet enhances selective uptake of LDL cholesteryl esters in the arterial wall. J Clin Invest.

[24] Ricci C, Ruscica M, Camera M, Rossetti L, Macchi C, Colciago A (2018). PCSK9 induces a pro-inflammatory response in macrophages. Sci Rep.

[25] Ruscica M, Ricci C, Macchi C, Magni P, Cristofani R, Liu J (2016). Suppressor of Cytokine Signaling-3 (SOCS-3) induces proprotein convertase subtilisin kexin type 9 (PCSK9) expression in hepatic HepG2 cell line. J Biol Chem.

[26] Jeong HJ, Lee HS, Kim KS, Kim YK, Yoon D, Park SW (2008). Sterol-dependent regulation of proprotein convertase subtilisin/kexin type 9 expression by sterol-regulatory element binding protein-2. J Lipid Res.

[27] Varghese JF, Patel R, Yadav UCS (2019). Sterol regulatory element binding protein (SREBP) -1 mediates oxidized low-density lipoprotein (oxLDL) induced macrophage foam cell formation through NLRP3 inflammasome activation. Cell Signal.

[28] Ridker PM, Everett BM, Thuren T, MacFadyen JG, Chang WH, Ballantyne C, et al; CANTOS Trial Group (2017). Antiinflammatory therapy with canakinumab for atherosclerotic disease. N Engl J Med.

[29] Jin K, Liu Y, Shi Y, Zhang H, Sun Y, Zhangyuan G (2020). PTPROt aggravates inflammation by enhancing NF-κB activation in liver macrophages during nonalcoholic steatohepatitis. Theranostics.

[30] Feng X, Sureda A, Jafari S, Memariani Z, Tewari D, Annunziata G (2019). Berberine in cardiovascular and metabolic diseases: From mechanisms to therapeutics. Theranostics.

