# NLRP3 inflammasome *via* IL-1β regulates PCSK9 secretion: Erratum

**DOI:** 10.7150/thno.68759

**Published:** 2022-01-01

**Authors:** Zufeng Ding, Xianwei Wang, Shijie Liu, Sichang Zhou, Rajshekhar A Kore, Shengyu Mu, Xiaoyan Deng, Yubo Fan, Jawahar L Mehta

**Affiliations:** 1 Henan Key Laboratory of Medical Tissue Regeneration, Xinxiang Medical University, Xinxiang, China; 2 Central Arkansas Veterans Healthcare System and Department of Internal Medicine, University of Arkansas for Medical Sciences, Little Rock, AR.; 3 Department of Neurological Surgery, Weill Cornell Medicine, New York; 4 Department of Pharmacology and Toxicology, College of Medicine, University of Arkansas for Medical Sciences, Little Rock; 5 Key Laboratory for Biomechanics and Mechanobiology of Ministry of Education, School of Biological Science and Medical Engineering, Beihang University, Beijing, China.; 6 Beijing Advanced Innovation Center for Biomedical Engineering; Beihang University, Beijing, China.

The authors regret that the original version of this paper 1 unfortunately contained some mistakes and some incorrect information for the author affiliations; and attribution of the grants to the appropriate PI was not included. The author affiliations are corrected as above. And the attribution of the grants to the appropriate PI is shown below. The authors apologize for any inconvenience that these errors may have caused.

## Attribution of the grants to the appropriate PI in Acknowledgments

This study was supported by funding from National Natural Science Foundation of China (No. 11772036 to Dr. Xiaoyan Deng, 11421202 to Dr. Yubo Fan) and National Key Research and Development Program in China (No. 2016YFB1101100 to Dr. Yubo Fan). Other funds were obtained from Grant-in-Aid from the Department of Veterans Affairs, Veterans Health Administration, Office of Research and Development, Biomedical Laboratory Research and Development, Washington, DC (No. I01BX000282 to Jawahar L Mehta). Additional support was provided by Stebbins Chair in Cardiology funds to Jawahar L Mehta.
