# Supplementary material for: NLRP3 inflammasome via IL-1β regulates PCSK9 secretion
Source: Theranostics. 2020 May 30;10(16):7100–10. doi: 10.7150/thno.45939 (PMC7330863; doi:10.7150/thno.45939)

**Supplemental Figure 1.** (**A**) Gene knockout validation in different mice. (B) PCSK9 expression in a variety of tissues from wild-type (WT) and several gene deletion mice groups. (**C**) Effect of insulin on PCSK9 expression. Western blots shown are representative of 3-5 independent experiments.

**Supplemental Figure 1**

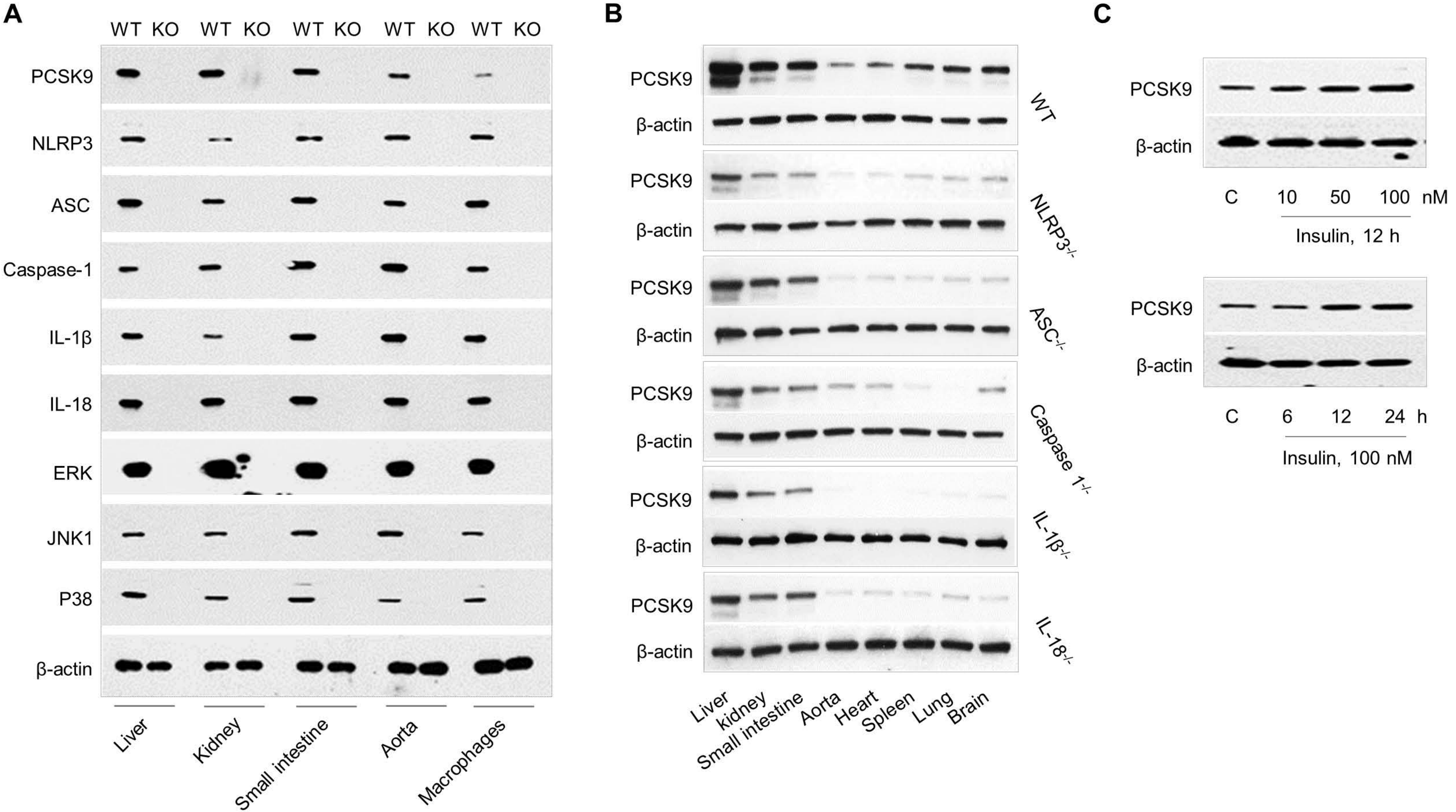

Supplement: Supplementary file 1 — Supplementary figures and tables. [file thnov10p7100s1.pdf]
